# 

**Published:** 1883-12

**Authors:** 


### CONTENTS.

### EXTRACTS.

Clinical Remarks on Dysmenorrhœa and Sterility ........ 65
Cow's Milk for Infants ........ 72
National Traits in Science ........ 77
Substitute for Mercurial Ointment ........ 80
Salicylic Acid in Typhoid Fever ........ 81
Human Obesity ........ 81
The Use of the Bromide Salts for Abdominal Neuroses ........ 82

### EDITORIAL.

Practical Chemistry for Students and Physicians—Chapter Sixth ........ 83
Exercise Questions on Chapter Sixth ........ 88
BOOKS ........ 89
PAMPHLETS ........ 96

